# Synaptic and cellular profile of neurons in the lateral habenula

**DOI:** 10.3389/fnhum.2013.00860

**Published:** 2013-12-16

**Authors:** Frank J. Meye, Salvatore Lecca, Kristina Valentinova, Manuel Mameli

**Affiliations:** ^1^Institut du Fer à MoulinParis, France; ^2^INSERM, UMR-S 839Paris, France; ^3^Université Pierre et Marie CurieParis, France

**Keywords:** lateral habenula, synaptic transmission, AMPA receptors, GABA receptors, neuromodulators

## Abstract

The lateral habenula (LHb) is emerging as a crucial structure capable of conveying rewarding and aversive information. Recent evidence indicates that a rapid increase in the activity of LHb neurons drives negative states and avoidance. Furthermore, the hyperexcitability of neurons in the LHb, especially those projecting to the midbrain, may represent an important cellular correlate for neuropsychiatric disorders like depression and drug addiction. Despite the recent insights regarding the implications of the LHb in the context of reward and aversion, the exact nature of the synaptic and cellular players regulating LHb neuronal functions remains largely unknown. Here we focus on the synaptic and cellular physiology of LHb neurons. First, we discuss the properties of excitatory transmission and the implications of glutamate receptors for long-term synaptic plasticity; second, we review the features of GABAergic transmission onto LHb neurons; and finally, we describe the contribution that neuromodulators such as dopamine (DA) and serotonin may have for LHb neuronal physiology. We relate these findings to the role that the LHb can play in processing aversive and rewarding stimuli, both in health and disease states.

## Introduction

Significant evidence is converging to the idea that the lateral habenula (LHb) strongly controls midbrain targets including the ventral tegmental area (VTA), substantia nigra pars compacta and the raphe nuclei (Christoph et al., 1986; Hikosaka, 2010). Functionally, the LHb contributes to the encoding of aversion and reward, and also plays a role in associated pathological conditions such as mood disorders and drug addiction (Hikosaka, 2010). A comprehensive understanding of LHb functions and their implications in neuropsychiatric disorders requires the dissection of the cellular and synaptic properties of neurons in this nucleus. Here, we describe how LHb neurons activity can be influenced by excitatory and inhibitory synapses and by neuromodulators, and we discuss the significance of this in relation to the potential role of the LHb in encoding rewarding and aversive stimuli, and in associated psychiatric disorders.

## The lateral habenula (LHb): a highway to the midbrain for processing aversion and reward

### Anatomical and morphological organization

**Figure 1:**
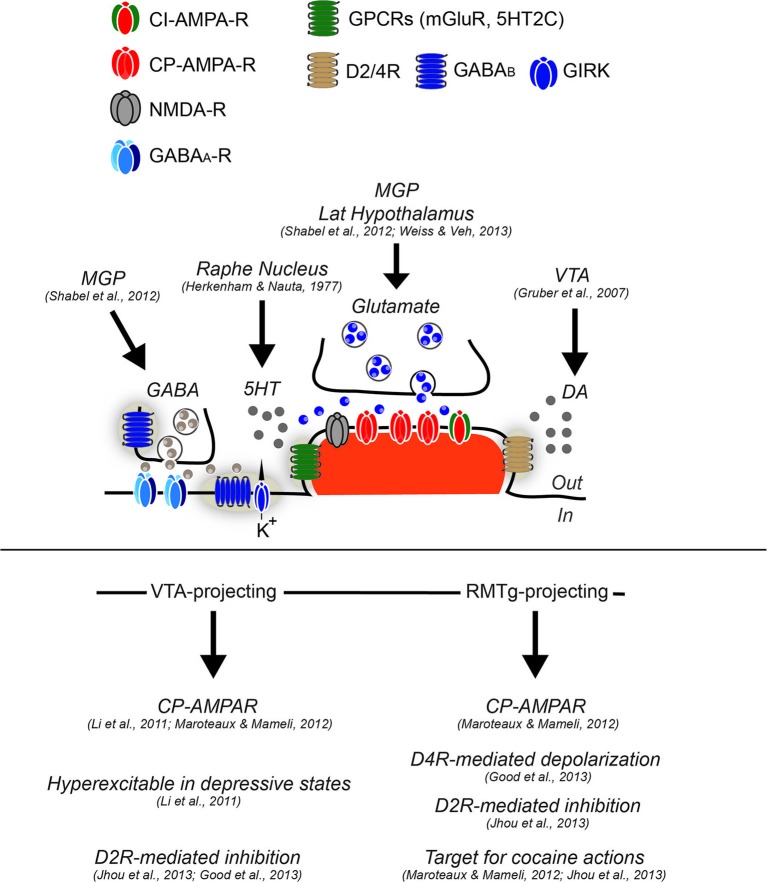
**Synaptic and cellular properties of LHb neurons.** Neurons in the LHb have been generally described as glutamatergic and projection type. While some feature of excitatory synapses have been described, functional evidence regarding GABAergic transmission or other type of regulatory mechanisms remain elusive, although functional GABA inputs arise from the medial globus pallidus (MGP). Glutamate transmission (arising from MGP and lateral hypothalamus) largely relies on α-Amino-3-hydroxy-5-methyl-4-isoxazolepropionic acid receptors (AMPA-R) lacking the subunit GluA2 and therefore calcium permeable (CP-AMPARs), although calcium-impermeable (CI-AMPARs) are likely to be expressed since the degree of inward rectification of AMPA-mediated excitatory postsynaptic currents (AMPA-EPSCs) is not complete (Li et al., 2011; Maroteaux and Mameli, 2012). The presence of mGluR have not yet been tested, however in situ hybridization images from the Allen Brain Atlas would suggest this. Conversely, LHb neurons have been shown to express postsynaptically dopamine 2 receptor and dopamine 4 receptors (D2R and D4R) sensing DA, likely released from the VTA, and serotonin receptor 2C (5HT2C) sensing serotonin released from dorsal raphe. GABA_A_ mediated transmission has been demonstrated to be functional, although the expression of GABA_A_ subtypes in this structure remains elusive. The GABA_B_ type of receptors has been shown to be expressed at least postsynaptically, however their coupling to GIRKs or other effectors remain to be established. LHb neurons projecting to VTA, and RMTg present cellular differences. While the VTA-projecting are hyperexcitable in depressive states, the RMTg projecting, are more sensitive to cocaine-evoked plasticity, and further present a dopamine-mediated depolarization through D4R activation. VTA and RMTg projecting neurons, seem to both express functional D2R, that if activated provide an hyperpolarization that decrease neuronal excitability.

The LHb, together with the medial habenula (MHb), is part of the epithalamus, and located above the thalamus at its posterior end close to the midline. The LHb comprises a medial and a lateral division (Andres et al., 1999; Hikosaka, 2010). LHb neurons present a dendritic arborization with postsynaptic spines (Figure 1; Weiss and Veh et al., 2011; Maroteaux and Mameli, 2012). Morphological and immunohistochemical studies revealed heterogeneous populations randomly distributed throughout the LHb based on somatodendritic organization and receptor expression (Weiss and Veh et al., 2011; Aizawa et al., 2012).

The main output of the LHb is glutamatergic (Li et al., 2011; Aizawa et al., 2012; Lammel et al., 2012; Stamatakis and Stuber, 2012). Axons from the LHb descend through the fasciculus retroflexus to deep structures: to GABAergic and dopaminergic neurons in the VTA, to GABAergic and serotoninergic neurons in the dorsal and median raphe, and to GABAergic neurons in the rostromedial tegmental nucleus (RMTg or tail-VTA; Herkenham and Nauta, 1979; Jhou et al., 2009; Barrot et al., 2012). However, single cell morphology studies also indicate that some LHb neurons can project to neighboring neurons within the LHb (Weiss and Veh et al., 2011), suggesting an internal control within the structure.

### LHb in aversion and reward

The LHb is pivotal in processing aversive and rewarding information. Delivery of an unexpected airpuff, a cue that predicts its onset, or even the omission of an expected reward, leads to a strong increase in the activity of LHb neurons in monkeys. Conversely, unexpected delivery of rewards and cues predicting a reward decrease LHb neuron firing. The LHb is inhibited more strongly as expected reward probability or magnitude increase (Matsumoto and Hikosaka, 2007, 2009). Importantly, the activity in LHb neurons is the inverse of that of dopamine (DA) neurons in the midbrain during aversive and rewarding states. The punishment-driven increase in excitation of LHb occurs earlier than that of DA neurons, suggesting the upstream control of the LHb on the DA system. This may not be reflected in the case of reward delivery, leaving the causality of this relationship still as an open question (Matsumoto and Hikosaka, 2007). Together these findings suggest that LHb neurons code for discrepancies between reward or punishment-related expectation and outcome.

Recent advances have shed light on the implication of LHb in driving aversive behaviors. Indeed, optogenetic activation of excitatory projection to the LHb, and LHb terminals in the midbrain drives aversive behaviors (Lammel et al., 2012; Shabel et al., 2012; Stamatakis and Stuber, 2012). However, while such evidence identified a crucial role of LHb in driving motivation, a precise dissection of synaptic and cellular properties within the LHb remains elusive and necessary to globally understand the role of this structure for specific behaviors.

## Synaptic transmission in the lateral habenula (LHb)

### Excitatory transmission in the lateral habenula (LHb)

LHb neurons receive glutamatergic projections from various structures that include the MGP, as well as the lateral hypothalamus, the cortex and likely the VTA (Weiss and Veh et al., 2011; Hnasko et al., 2012; Shabel et al., 2012). Below, we will discuss the postsynaptic receptors that mediate excitatory transmission onto LHb neurons and their role in long-term and experience-dependent synaptic plasticity.

#### Glutamate receptors

The predominant receptor in the LHb mediating fast excitatory transmission is the AMPA-type glutamate receptor (AMPAR): a heterotetrameric complex highly sensitive to experience-driven changes (Hollmann et al., 1994). The AMPA GluA2 subunit is edited at the mRNA level (glutamine to arginine) (Lomeli et al., 1994) conferring channel impermeability to calcium. AMPARs lacking the subunit GluA2 are instead calcium permeable (CP-AMPARs), and exhibit inward rectification due to voltage-dependent block of the receptor by intracellular polyamines at positive potentials (Burnashev et al., 1992; Donevan and Rogawski, 1995). Patch-clamp recordings in rats and mice from LHb neurons indicate that the AMPA-mediated currents are inwardly rectifying, suggesting that glutamatergic input to the LHb relies, at least to a significant extent, on CP-AMPARs (Figure 1; Li et al., 2011; Maroteaux and Mameli, 2012). CP-AMPAR expression is a general feature of LHb neurons, as projection-specific retrograde labeling demonstrated that the rectification index, a measure reflecting the presence of GluA2-lacking AMPARs, did not differ between LHb subpopulations (Figure 1; Li et al., 2011; Maroteaux and Mameli, 2012).

Metabotropic glutamate receptors (mGluRs) generally strongly modulate CP-AMPAR expression. Indeed, mGluRs trigger long-term depression (LTD) of AMPAR currents specifically when CP-AMPARs are present (Luscher and Huber, 2010). In several structures including the VTA, the nucleus accumbens and the cerebellum, mGluR activation triggers a switch from CP-AMPARs to calcium impermeable AMPARs. mGluRs evoke CP-AMPAR internalization, regulate experience-induced synaptic plasticity, and in some cases restore experience-driven adaptations (Bellone and Luscher, 2006; Kelly et al., 2009; Clem and Huganir, 2010; McCutcheon et al., 2011). mGluRs are also expressed in the LHb, although seemingly at relatively low levels (Figure 1). However, the function of mGluRs in the LHb and their relationship with the CP-AMPARs in this structure remains unknown.

Interestingly, the NMDA-mediated component at LHb synapses at positive potentials is small compared to the one mediated by AMPA receptors, indicative for a low expression of synaptic N-methyl-d-aspartate receptors (NMDAR; Li et al., 2011; Maroteaux and Mameli, 2012). Whether NMDARs are or not expressed at excitatory synapses, or whether their expression is limited to extrasynaptic sites remains to be established. Further insight in their subunit composition, and their potential role in long-term synaptic plasticity also still needs to be addressed.

#### Synaptic plasticity in the LHb

CP-AMPARs regulate the induction of several forms of long-term synaptic plasticity (Kullmann and Lamsa, 2011). In the VTA, CP-AMPAR expression, triggered by acute cocaine exposure (Bellone and Luscher, 2006), allows the expression of a CP-AMPAR-dependent long-term potentiation (LTP) that relies on postsynaptic hyperpolarization (Mameli et al., 2011). Such a CP-AMPAR-dependent LTP, relying on postsynaptic hyperpolarization, has also been described for interneurons of the hippocampus (Lamsa et al., 2007; Le Roux et al., 2013). Whether CP-AMPARs also drive long-term plasticity in the LHb has been recently explored. Stimulation of excitatory presynaptic terminals paired with postsynaptic hyperpolarization leads to an LTD of AMPAR currents (Maroteaux and Mameli, 2012). Interestingly, exposure to cocaine for 2 consecutive days strengthens CP-AMPAR transmission onto LHb neurons that project to the RMTg, but not onto those that project to the VTA. Moreover, the cocaine exposure switches the direction of long-term plasticity from LTD to LTP in these RMTg-projecting neurons. It remains to be elucidated whether and how these LHb neurons can be hyperpolarized in physiological conditions to allow CP-AMPARs to be efficient. Interestingly, stimulation of the main fiber bundle converging to the LHb (i.e., the stria medullaris) drives a strong hyperpolarization that reduces neuronal activity representing an ideal state for CP-AMPAR activation (Chang and Kim, 2004). The receptors mediating an hyperpolarizing state may be several, and below we will discuss the potential implication of some of them including GABA_A_Rs, GABA_B_Rs, and D2Rs. Dynamics of AMPARs in the LHb have been recently implicated in depressive-like states. Indeed, evidence suggests that Calcium-calmodulin-dependent protein kinase II (βCaMKII)-mediated trafficking of GluA1-containing AMPARs in LHb may participate in the expression of depressive-like symptoms (Li et al., 2013). Altogether these results place maladaptations of AMPAR transmission in the LHb as a potential cellular substrate for psychiatric disorders associated with reward or aversion.

### Inhibitory transmission in the lateral habenula (LHb)

The LHb receives strong GABAergic innervation (Araki et al., 1984), presumably coming from long-range projections, since local interneurons are largely absent (Smith et al., 1987; Li et al., 2011). One GABAergic input arises from the MGP, and preferentially targets the lateral portion of the LHb (Shabel et al., 2012). Anatomical studies suggest that other GABAergic projections may arise from the diagonal band of broca, the lateral preoptic area, the nucleus accumbens, substantia innominata, and the ventral pallidum (Geisler and Trimble, 2008), although their functional properties remain unknown. Evidence suggests that GABAergic transmission in the LHb is mediated by both GABA_A_Rs and GABA_B_Rs, and we will discuss their implications for LHb neuronal function.

#### GABA_A_ receptors

GABA_A_Rs are pentameric ionotropic receptors, assembled out of a larger available pool of 19 subunits, which determine the conduction kinetics of the receptor as well as its affinity for GABA (Farrant and Nusser, 2005). In mice the LHb harbors mRNA (and largely also expresses the protein) for α1–3 (but not α4–6), β1 (but not β2–3) and γ1–2 subunits (but not δ subunits) although unmentioned subunits were not assessed (Hortnagl et al., 2013). Another study conducted in rat tissue yielded slightly different results, indicating that GABA_A_Rs in the LHb mainly consist of α1, β2 and γ3 subunits. These data are in line with a synaptic, rather than an extrasynaptic localization of GABA_A_Rs (Figure 1; Pirker et al., 2000; Hortnagl et al., 2013). Evidence suggests that strong inhibitory GABAergic signaling via the GABA_A_ receptor takes place in the LHb. First, the LHb plentifully contains the potassium chloride cotransporter-2 (KCC2), the main chlorine-extrusion mechanism ensuring that GABA_A_R signaling is inhibitory. Second, bath application of exogenous GABA in a slice preparation elicits large GABA_A_R-mediated currents in LHb neurons. Finally GABA_A_R-mediated miniature inhibitory spontaneous currents were observed in the LHb, indicating synaptic GABA_A_R-activation upon single vesicle release (Wang et al., 2006).

GABAergic signaling within the LHb has relevance in the context of drug intake. GABA (but not glutamate) immunolabeling decreased in the LHb in rats withdrawn (5 days) from chronic treatment with cocaine (Meshul et al., 1998). Prolonged cocaine withdrawal (21 days) increased binding of a radiolabeled benzodiazepine (which binds to GABA_A_Rs) in the whole LHb, whereas acute withdrawal from this treatment slightly decreased binding (Keys and Ellison, 1999). Furthermore, withdrawal from amphetamine yielded bidirectional differences in GABAergic markers in the LHb, depending on drug concentration (Yin et al., 2012). These findings indicate that drastic adaptations occur in GABAergic innervation of the LHb upon psychostimulant exposure. (Keys and Ellison, 1999) suggest that diminished GABAergic transmission in the LHb may stand at the basis of neurotoxicity in the fasciculus retroflexus, leading to a loss of inhibitory control from LHb over monoaminergic systems.

#### GABA_B_ receptors

GABA transmission also relies on G_i/o_ protein-coupled GABA_B_Rs, which are responsible for slower and late inhibitory conductance (Hill, 1985). GABA_B_Rs assemble into heteromers composed of a GABA_B_1 (with distinct variants GABA_B_1_A_ and GABA_B_1_B_) and a GABA_B_2 subunit, which are required for normal receptor functioning (Marshall et al., 1999; Bettler et al., 2004). Ultrastructural studies show that GABA_B_R subunits can generally be present both on pre- and postsynaptic sites as well as on extrasynaptic membranes (Figure 1; Lujan and Ciruela, 2012). The habenular complex (comprising both the MHb and LHb) is among the regions with the highest expression of GABA_B_Rs. However, the functions of GABA_B_Rs in these nuclei in both physiological and pathological conditions remain unknown (Margeta-Mitrovic et al., 1999; Liang et al., 2000). Within the habenular complex, the LHb present a fairly high expression of GABA_B_Rs, although less than in the MHb and no obvious differences are present in the two GABA_B_1Rs variants (Liang et al., 2000).

GABA_B_R activation inhibits adenylyl cyclase and mediates the hyperpolarization of post-synaptic membranes by activation of inwardly rectifying potassium (GIRK) channels (Luscher et al., 1997). Interestingly, a moderate expression of Kir3.2 potassium channel subunit has been detected in the LHb (Geisler et al., 2003). Indeed, activation of GABA_B_Rs by the selective agonist baclofen evoked an outward postsynaptic current in LHb neurons recorded (Wang et al., 2006) and together with immunocytochemistry evidence, this idicates the expression of functional postsynaptic GABA_B_Rs in the LHb (Geisler et al., 2003). GABA_B_Rs activation controls a broad amount of neuronal properties including excitability and synaptic strength (Luscher and Slesinger, 2010). The potential role of GABA_B_Rs in controlling LHb neurons functions, especially in the context of reward and aversion and related pathologies, remains to be evaluated. Dysregulation of GABA transmission and GABA_B_ function has been implicated in various central nervous system (CNS) disorders including anxiety, depression and addiction where the role of LHb is crucial (Hikosaka, 2010; Luscher and Slesinger, 2010).

### Neuromodulatory systems in the lateral habenula (LHb)

#### Dopamine (DA) modulation in the LHb

Dopaminergic nuclei such as the VTA and substantia nigra pars compacta receive input from the LHb, and also provide feedback projections, suggesting that DA may modulate LHb activity (Phillipson and Pycock, 1982; Gruber et al., 2007). Indeed, both the local application of DA and the systemic administration of dopaminergic agonists increase firing of LHb neurons (Kowski et al., 2009). Moreover, tetanic stimulation of the VTA increases LHb neuron firing rate (Shen et al., 2012). Conversely, *in vivo* recordings show that in LHb neurons activated by a painful stimulus, the** single-pulse stimulation of VTA and substantia nigra inhibits the firing of ~90% of the LHb neurons (Shen et al., 2012). Together this suggests a complex role of the midbrain and DA in controlling activity of LHb neurons.

Fibers expressing tyrosine hydroxylase, the rate-limiting enzyme in the synthesis of DA, have been demonstrated in the LHb (Geisler et al., 2003; Gruber et al., 2007; Aizawa et al., 2012). D2Rs and D4Rs are also functionally expressed in this region (Aizawa et al., 2012; Good et al., 2013; Jhou et al., 2013; Figure 1). Acting on the D2Rs, both DA and DA receptor agonists induced an hyperpolarization that drives a decrease in firing frequency in LHb neurons projecting to both RMTg and VTA (Figure 1; Good et al., 2013; Jhou et al., 2013). On the other hand DA binding to D4Rs depolarizes LHb neurons that preferentially project to the RMTg (Figure 1; Good et al., 2013). Cocaine evokes an overall excitation of LHb neurons projecting to RMTg, which has been shown to contribute to aversive conditioning after the drug rewarding effects has faded out, consistent with the theory of opponent processes (Solomon and Corbit, 1974; Jhou et al., 2013). The cellular mechanisms underlying this late onset cocaine-evoked excitation are unknown. Altogether, this evidence suggests that DA signals from the midbrain control LHb neuronal activity, providing new insights for the behavioral relevance of this feedback connection. Moreover, two notable points must be kept in mind. First, dopaminergic innervation of the LHb may also, to a lesser extent, come from other regions than the midbrain, such as the posterior hypothalamus and the periaqueductal gray (Gruber et al., 2007). Second, midbrain nuclei may not just use DA to regulate the LHb. Indeed VTA neurons with a glutamatergic profile also innervate the whole LHb (Hnasko et al., 2012), suggesting complex regulatory feedback from the midbrain to LHb that needs to be explored further.

#### 5HT modulation in the LHb

Analogously to the DA system, there is evidence that LHb neurons powerfully modulate raphe serotonin neurons, but also receive a serotoninergic feedback signal (Figure 1; Wang and Aghajanian, 1977; Mengod et al., 1990; Aizawa et al., 2012). The modulation by serotonin in the LHb neurons was recently investigated. By using optogenetic tools to input-specifically activate AMPARs at the MGP-to-LHb pathway, it was shown that serotonin bath application decreases AMPA-mediated transmission likely via a presynaptic rather than a postsynaptic mechanism (Shabel et al., 2012). These results indicate that either serotonin receptors in the LHb are exclusively presynaptic, or that they exert their effect through postsynaptic receptors, yet to be identified (Figure 1), and a retrograde messenger. Instead, this configuration may represent an input- or region-specific property since MGP inputs only innervate the lateral portion of the LHb (Hong and Hikosaka, 2008; Shabel et al., 2012). In this context, it is important to note that, while stimulation of the MGP-LHb pathway in behaving mice leads to avoidance behavior, the implications of the serotoninergic component in this behavior remains unknown.

## Concluding remarks

Initial evidence collected in the early 1980’s pointed to the potential importance of the LHb in controlling midbrain structures. However, only much more recently it is starting to become clear that this structure participates in the encoding of aversive and rewarding stimuli, influences motivational states, and contributes to pathologies such as mood disorders and addiction. These findings highlight the importance to understand how LHb neuronal activity levels are influenced by the integration of local synaptic and neuromodulatory signals.

While we have discussed the presumably key input signals and their receptors in this review, tentative evidence suggests that other potentially relevant molecules may also act in the LHb, by mechanisms that remain largely unknown. Among these potential modulatory signals are orexins (Peyron et al., 1998), acetylcholine (Geisler et al., 2003), vasopressin (Rood et al., 2008), substance P (Yang et al., 2013), and BDNF (Liu et al., 2001). Another notable point is that LHb neuronal activity will also largely be dependent on a variety of important intrinsic ion channels that LHb neurons express, which were beyond the scope of the current review. For instance, LHb neurons projecting to the VTA and the raphe nuclei express T- and L-type calcium channels that allows for long-lasting neuronal discharges as well as hyperpolarization-activated cyclic nucleotide-gated cation channels (HCN) suggesting an autonomous pacemaker activity (Chang and Kim, 2004; Poller et al., 2011).

The big challenge in this field is now to identify which synaptic and intrinsic properties adapt during or after exposure to aversion or reward; and to understand the downstream implications of such changes on monoamines like the DA and serotonin systems. Such a framework may represent the basis to understand the maladaptive mechanisms taking place in neuropsychiatric disorders where the activity of the LHb is altered, and may offer a window to further develop therapeutic strategies.

## Conflict of interest statement

The authors declare that the research was conducted in the absence of any commercial or financial relationships that may represent a potential conflict of interest.
